# From mechanism to therapy: advances in macrophage polarisation and targeted intervention in osteosarcoma

**DOI:** 10.3389/fimmu.2026.1826432

**Published:** 2026-05-20

**Authors:** Haojun Huang, Defeng Dong, Huayan You, Xiaoling Fu

**Affiliations:** 1 School of Nursing, Yanbian University, Yanji, Jilin, China; 2 School of Graduate Education, Shandong Sport University, Jinan, Shandong, China; 3 Hunan Key Laboratory of Medical Epigenomics & Department of Dermatology, The Second Xiangya Hospital of Central South University, Changsha, Hunan, China; 4 Department of Orthopaedics, The Second Affiliated Hospital of Nanchang University, Nanchang, Jiangxi, China

**Keywords:** immunotherapy, osteosarcoma, polarisation, tumour microenvironment, tumour-associated macrophages

## Abstract

Osteosarcoma is the most common type of primary malignant bone tumour in children and adolescents. Despite advances in surgery and neoadjuvant chemotherapy, tumour metastasis and recurrence are still the main factors affecting patient prognosis. Recent research has shown that immune cells in the tumour microenvironment (TME), particularly tumour-associated macrophages (TAMs), play a crucial role in osteosarcoma progression. Macrophages exhibit high plasticity and can differentiate into the M1 type, which has anti-tumour functions, or the M2 type, which promotes tumour development. This paper provides a systematic review of the research on TAMs and their mechanisms in promoting osteosarcoma progression by driving tumour cell proliferation, mediating immune evasion and facilitating metastasis and angiogenesis. Additionally, it provides a systematic outline of potential therapeutic strategies for inhibiting osteosarcoma development by targeting and regulating macrophage polarisation. These strategies include drug interventions, nanotechnology and combined immunotherapy. This paper aims to elucidate the key mechanisms by which macrophage polarisation regulates the biological behaviour of osteosarcoma. This will provide a theoretical basis and strategic references for the precise immunotherapy of osteosarcoma.

## 1 Introduction

Osteosarcoma (OS) is a highly malignant primary bone tumour that most commonly arises in the metaphysis of long bones in adolescents. It is characterized by its aggressive nature and high propensity for lung metastasis (1). Although multidisciplinary treatment, particularly surgery combined with neoadjuvant chemotherapy, has improved outcomes for patients with localised disease, the prognosis for advanced or metastatic osteosarcoma remains poor, with a five-year survival rate of less than 30%. The aggressive and metastatic nature of the disease continues to present major challenges in clinical treatment (2).

The role of the TME in the occurrence and development of osteosarcoma has received increasing attention in recent years. TAMs are among the most abundant immune cells in the TME and play a dual role in regulating tumour progression through phenotypic plasticity. Classically activated M1 macrophages exert anti-tumour effects by secreting pro-inflammatory factors and presenting tumour antigens, while alternatively activated M2 macrophages promote tumour progression through immunosuppressive and pro-angiogenic functions (3). Research indicates that the level of M2-like TAM infiltration is reported to be higher in metastatic/relapsed osteosarcoma tissue, while the proportion of M1-like TAM is markedly lower. This suggests that an imbalance in the polarisation of macrophages is a critical driving factor in the progression of osteosarcoma (4).

Previous studies have examined the role of macrophages within the osteosarcoma microenvironment, proposing therapeutic strategies that target the reprogramming of macrophages. However, these studies have primarily focused on the general functions of macrophages or single polarisation regulation strategies. Furthermore, given the rapid development of emerging strategies such as nanotechnology, sonodynamic therapy and metabolic regulation, the potential applications of macrophage-targeted therapy in the treatment of osteosarcoma are constantly evolving. It is therefore crucial to integrate the latest research findings to inform future developments. More importantly, accumulating evidence indicates that macrophage polarisation is not regulated by isolated signalling pathways, but rather by a dynamic and context-dependent regulatory network, in which different pathways may exert synergistic or even opposing effects depending on the tumour microenvironment.

In this context, we propose that TAM polarisation in osteosarcoma can be conceptualised as a dynamic equilibrium system characterised by a potential “tipping point”, at which cumulative regulatory inputs drive a shift from an anti-tumour M1-dominant state to a pro-tumour M2-dominant state. This transition is likely governed by the hierarchical dominance and cross-regulation of multiple signalling axes, including NF-κB, AMPK and JAK-STAT, whose functions may vary across different biological contexts. Operationally, this tipping point can be understood as the stage at which pro-tumour inputs, such as sustained IL-4/IL-13 or CSF-1 signalling, hypoxia, lactate accumulation, oxidative phosphorylation or fatty acid oxidation bias, and tumour-derived exosomal signals, outweigh antitumour inflammatory cues such as IFN-γ, GM-CSF, TLR activation and NF-κB-mediated acute inflammatory activation. Rather than representing a single molecular switch, the tipping point may therefore be reflected by a coordinated change in measurable parameters, including an increased CD163/CD206/Arg1 or IL-10/TGF-β signature, reduced CD86/iNOS or TNF-α/IL-12 expression, impaired phagocytic and antigen-presenting capacity, enhanced angiogenic and matrix-remodelling activity, and spatial enrichment of immunosuppressive TAMs in hypoxic or invasive tumour regions. From this perspective, the model can be operationalised in experimental and clinical studies by integrating temporal cytokine profiling, macrophage marker panels, metabolic readouts, single-cell transcriptomic states and spatial localisation patterns, rather than relying on a single M1 or M2 marker. Recognising these context-dependent contradictions is essential for moving beyond descriptive summaries towards a more integrated and mechanistic understanding of TAM-mediated tumour progression.

This review summarizes the molecular mechanisms by which TAMs contribute to osteosarcoma progression, focusing on proliferation, immune evasion, metastasis and angiogenesis. It incorporates a multi-layered regulatory network that encompasses cytokines, signalling pathways, exosomes and metabolic reprogramming. In terms of treatment strategies, we categorise intervention strategies targeting macrophage polarisation innovatively into three types: repolarising M2-like TAM to M1-like, inducing the polarisation of resident TAM to M1-like, and inhibiting the conversion of M1-like to M2-like. Importantly, rather than treating these strategies as equivalent, this review interprets them within the broader regulatory framework described above, highlighting their mechanistic convergence, context dependence and potential limitations. This approach encompasses not only traditional drugs, such as CSF-1R inhibitors and natural products, but also emerging technologies, such as nanomaterials, sonodynamic therapy, photothermal therapy and cytokine recycling systems (CERS). This demonstrates the promising prospects of interdisciplinary treatment.

Overall, this review aims to provide an integrated perspective on macrophage polarisation in osteosarcoma and its therapeutic implications. By synthesising current evidence and highlighting unresolved inconsistencies, we seek to offer a more critical and forward-looking framework for the development of precise immunotherapeutic strategies in osteosarcoma.

## 2 The overview of osteosarcoma

Osteosarcoma, a tumour originating from mesenchymal cells, accounts for 17% of primary bone tumours and 42% of malignant bone tumours (5), making it the most common malignant primary bone tumour in adolescents (6). Early symptoms of osteosarcoma commonly include localised pain, swelling and joint dysfunction, all of which progressively worsen. In severe cases, pathological fractures may occur (7). Osteosarcoma usually develops in the metaphysis of long bones, such as the distal femur, proximal tibia and proximal humerus. It is characterised by high invasiveness and a poor prognosis (8). Research indicates that the overall survival rate for osteosarcoma after five years is between 60% and 70%. However, this rate falls to below 30% in patients who experience metastasis. Haematogenous spread is the typical mode of metastasis, with the lungs being the most common site (9).

Over the past few decades, the primary treatment strategy for osteosarcoma has been a combination of surgical intervention and neoadjuvant chemotherapy (10). Nevertheless, the recurrence of disease and pulmonary metastasis continue to present significant challenges in the clinical management of osteosarcoma. In recent years, immunotherapy has emerged as a novel treatment approach that has achieved significant success in treating various tumours by targeting and modulating the immune microenvironment (11). However, due to the high heterogeneity of the tumour immune microenvironment in osteosarcoma, TAMs, which are present in large numbers, have been confirmed to promote osteosarcoma progression and lung metastasis. They are also associated with a poor prognosis, which limits the efficacy of immunotherapy in osteosarcoma (12, 13). Therefore, exploring the potential mechanisms related to macrophages in the progression of osteosarcoma and establishing novel, effective treatment strategies has become urgent.

## 3 Macrophage polarisation and systems-level regulation in osteosarcoma

### 3.1 The origins and fundamental characteristics of macrophages

The TME is a complex network comprising tumour-infiltrating immune cells, mesenchymal stem cells, tumour cells and inflammatory mediators (14). TAMs are the most common type of immune cell found in the TME (15). Macrophages originate mainly from haematopoietic stem cells or monocytes, and are recruited to tissues for immune surveillance and response (16). Recent studies have found that tissue-resident macrophages can originate from the yolk sac and foetal liver in the early stages of embryonic development, before HSCs emerge. These macrophages can then undergo self-renewing proliferation (17). Therefore, macrophages can be categorised into two main groups: tissue-resident macrophages, which are derived from the embryo, and blood monocytes, which originate from the bone marrow (18). Macrophages are highly plastic and regulate innate immunity via phagocytosis, antigen presentation, and cytokine production (19).

TAM exhibit a high degree of phenotypic heterogeneity and functional plasticity in the immune microenvironment. Macrophages can be classified as either classical activated M1 type TAMs or alternatively activated M2 type TAMs, based on their activation status and functions (20). M1-like TAMs are generally associated with antitumour functions, while M2-like TAMs are linked to immunosuppressive and pro-angiogenic roles (21).

This binary classification is a simplified framework, as TAM phenotypes exist along a functional spectrum influenced by dynamic microenvironmental cues. Rather than representing two discrete and mutually exclusive states, M1-like and M2-like macrophages should be viewed as the opposite ends of a continuous activation landscape, within which cells may adopt intermediate or hybrid phenotypes. Accumulating evidence from single-cell analyses indicates that TAMs frequently co-express canonical M1- and M2-associated markers, reflecting simultaneous engagement of inflammatory and immunoregulatory programmes rather than strict polarisation into one state.

Importantly, macrophage phenotypes also exhibit pronounced spatial and temporal heterogeneity within the tumour microenvironment. TAMs located in hypoxic tumour cores are more likely to display immunosuppressive, pro-angiogenic, and metabolically adapted features, whereas macrophages at the invasive margin may retain partial inflammatory activity or participate in tissue remodelling and immune cell recruitment. In addition, macrophage activation states are not static but evolve dynamically during tumour progression and in response to therapeutic interventions, including chemotherapy and immunotherapy.

In this review, while we retain the M1/M2 terminology for clarity and consistency with the existing literature, we interpret these categories as simplified reference points within a broader and dynamic continuum of macrophage activation states, rather than as fixed or exclusive phenotypes.

### 3.2 Signalling networks and dynamic regulation of TAM polarisation

Research has shown that, when stimulated by specific microenvironmental signals, unpolarised M0-type TAMs can polarise into either M1-like or M2-like TAMs. There can also be mutual conversion between the M1 and M2 phenotypes. Imbalances in M1/M2-like TAMs have been associated with tumour progression, but this relationship is context-dependent (22). High M2-like TAM infiltration is often reported in malignant tumours (23), although magnitude varies by model. In gastric cancer, the proportion of M2-like TAMs is significantly higher than in normal adjacent tissue, and related gene expression is increased (24). In contrast to cases of non-metastatic/non-recurrent osteosarcoma, cases of metastatic/recurrent osteosarcoma exhibit a significant decrease in the levels of both M0- and M1-like TAM, alongside a notable increase in the quantity of M2-like TAM. This suggests that the level of polarisation of M2-like TAMs may be associated with the progression of osteosarcoma disease (25).

**Figure 1:**
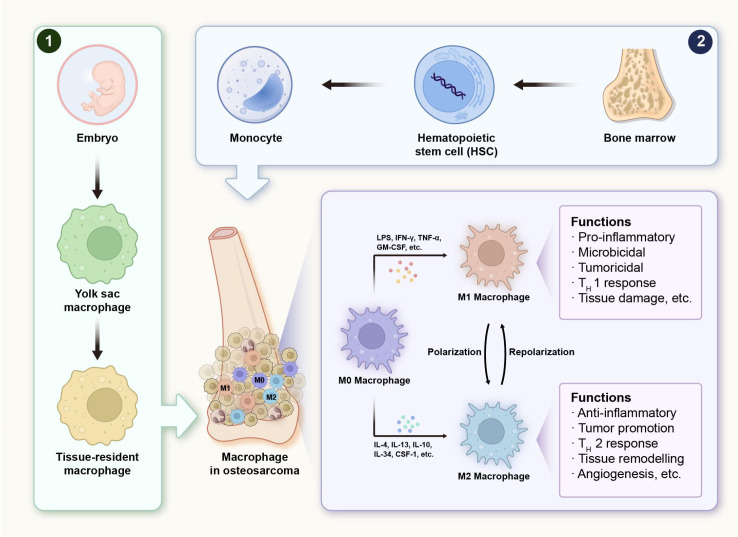
Schematic diagram of macrophage polarisation and regulatory mechanisms. .

The process of macrophage polarisation is precisely regulated by a multi-layered network involving cytokines, signalling pathways and transcription factors. M1-like TAMs are induced by Th1-type cytokines, such as IFN-γ (26), GM-CSF (27) and TNF-α (28). They exhibit strong antibacterial and antitumour activities. By contrast, M2-like TAMs are produced in response to Th2 cytokines such as IL-4 and IL-13. They play a role in tissue repair and inflammation suppression, as well as supporting tumour growth (29) (**Figure 1**). This plasticity enables context-dependent shifts, offering therapeutic opportunities to reprogram TAMs. Tumour cells can recruit monocytes to the TME and induce their differentiation into M2-like TAM by secreting factors such as colony-stimulating factor 1 (CSF-1) and interleukin 34 (IL-34) (30). In addition to tumour cells, TAM can secrete cytokines such as IL-4 and IL-13. These cytokines act on their own receptors via autocrine or paracrine mechanisms, thereby inducing type 2 polarisation (31). **Figure 1** highlights these canonical polarisation pathways and the plasticity of TAMs, illustrating how dynamic context-dependent cues can shift macrophages between M1- and M2-like states. This framework provides a simplified reference to interpret macrophage functional states while acknowledging that actual phenotypes exist along a continuous spectrum.

The polarisation of TAM is regulated by several signalling pathways, including the NF-κB, PI3K-AKT, MAPK, AMPK, CCL2-CCR2, JAK-STAT and AKT/FOXO1 pathways (32–35). The abnormal activation of the NF-κB pathway has been linked to various diseases, including cancer and chronic inflammation (36). In macrophages, pattern recognition receptors (PRRs) recognise damage-associated molecular patterns (DAMPs) or pathogen-associated molecular patterns (PAMPs), thereby leading to the activation of NF-κB via the classical pathway. Subsequently, this activation upregulates the expression of TNF-α, IL-6, interferons and inducible nitric oxide synthase (iNOS), which is classically associated with the induction of a pro-inflammatory, M1-like programme (37, 38). However, the role of NF-κB in macrophage polarisation is not unidirectional. Although NF-κB signalling drives M1-like TAM polarisation in response to stimuli such as lipopolysaccharide, several studies suggest that, under Th2-skewed conditions or in tumour-associated cytokine milieus, NF-κB activity may also participate in sustaining M2-associated functions or survival programmes (39). This apparent discrepancy indicates that NF-κB should not be interpreted as a simple binary switch, but rather as a context-dependent signalling node whose biological output depends on the nature, intensity and duration of upstream stimulation, as well as its interaction with other pathways within the tumour microenvironment. Activation of the PI3K-AKT pathway can promote M2-like metabolic reprogramming, including fatty acid oxidation, which provides energy for polarisation. Mechanistically, PI3K-AKT signalling interacts closely with mTORC1/2 and downstream metabolic regulators, coordinating glucose utilisation, lipid metabolism and mitochondrial function. This pathway not only supports the bioenergetic demands of M2-like macrophages but also influences transcriptional outputs through factors such as HIF-1α and IRF4, thereby linking metabolic adaptation with functional polarisation (40). Induction of IL-4 promotes activation of the MAPK pathway, which in turn drives the phosphorylation cascade of MK2. At a mechanistic level, MAPK signalling integrates extracellular cytokine cues with intracellular transcriptional regulation through ERK, JNK, and p38 modules, thereby modulating downstream transcription factors such as AP-1 and CREB. This promotes the sustained expression of M2-associated genes, including Arg-1, CD206 and IL-10, and contributes to the stabilisation of alternative activation programmes rather than merely initiating polarisation (41).

AMPK (AMP-activated protein kinase) is a serine/threonine kinase that is widely conserved in eukaryotes, and it plays a central role in the regulation of metabolic pathways. Its activation has been found to be crucial for metabolic adaptation and immune regulation within the tumour microenvironment. Studies indicate that AMPK activation can enhance glycolysis through the Akt-HIF1α-mTOR pathway, thereby promoting TAM polarisation towards the M1 phenotype (42). Nevertheless, the effect of AMPK on macrophage polarisation remains controversial and appears to be highly dependent on downstream signalling context. Jiang et al. found that, in the TME, metformin increases the proportion of M1-like TAMs and decreases the proportion of M2-like TAMs via the AMPK-NF-κB signalling pathway (43). However, Zhou et al.’s study found that metformin promotes M2 polarisation via the AMPK/PGC-1α/PPAR-γ signalling pathway (44). Rather than representing mutually exclusive conclusions, these findings suggest that AMPK may function as a metabolic rheostat, with its downstream effects shaped by cell type, cytokine exposure, nutrient availability and pathway crosstalk. Mechanistically, AMPK can differentially engage distinct downstream signalling branches, including mTOR inhibition, PGC-1α/PPAR-γ activation, and crosstalk with NF-κB and HIF-1α pathways, thereby linking cellular energy sensing to transcriptional and metabolic reprogramming. Th2-type cytokines, such as IL-4 and IL-13, can activate the AMPK pathway and upregulate the expression of M2-related markers, such as IL-10, thereby favouring M2 polarisation under specific microenvironmental conditions (45). In contrast, under conditions of acute inflammatory stimulation or metabolic stress, AMPK activation may cooperate with pro-inflammatory signalling axes to support glycolytic reprogramming and M1-like responses, highlighting its context-dependent functional plasticity. Taken together, the conflicting observations surrounding AMPK further support the view that macrophage polarisation is governed by an integrated and hierarchical regulatory network, rather than by any single pathway acting in isolation. This perspective positions AMPK not as a unidirectional regulator, but as a context-sensitive integrative node whose functional output depends on the balance between inflammatory and metabolic signalling inputs within the tumour microenvironment.

Furthermore, the CCL2-CCR2 chemokine axis plays a vital role in regulating the direction of polarisation. Activation of this axis can induce the differentiation of M2-like macrophages, whereas inhibiting its signalling enhances the expression of M1-related genes and cytokines (46). Additionally, signalling pathways such as JAK-STAT and AKT/FOXO1 play a key role in M2 polarisation, forming an interconnected regulatory network (47).

GATA3 (GATA-binding protein 3) is a key member of the GATA family. It regulates the expression of downstream genes by recognising the DNA sequence A/T GATA A/G. GATA3 is involved in various biological processes, including immune regulation, tumour formation, and organ development (48, 49). In macrophage polarisation, GATA3 can promote IL-4 production by activating the Notch signalling pathway, thereby driving macrophages to differentiate into the M2 phenotype. Conversely, GATA3 knockdown using shRNA can inhibit M2-like TAM polarisation (50). STAT3/6 and the nuclear receptor peroxisome proliferator-activated receptor gamma (PPARγ) are also important transcription factors that regulate M2 polarisation (51). STAT3 can be activated and translocated to the nucleus by cytokines such as IL-6 and IL-10. This promotes M2 polarisation by regulating signalling pathways, including JAK/STAT, PI3K/AKT and MAPK (52, 53).

### 3.3 Microenvironmental and metabolic regulation of TAM states

Metabolic reprogramming in macrophages should not be viewed merely as a consequence of polarisation, but rather as a central regulatory layer that actively drives functional state transitions. Pro-inflammatory, M1-like macrophages are typically characterised by enhanced aerobic glycolysis, increased pentose phosphate pathway activity, and disrupted tricarboxylic acid (TCA) cycling, leading to the accumulation of metabolites such as succinate and citrate. These metabolites can stabilise HIF-1α and promote the production of inflammatory mediators, including IL-1β and nitric oxide, thereby reinforcing inflammatory activation programmes (54).

In contrast, M2-like macrophages preferentially utilise oxidative phosphorylation, fatty acid oxidation, and mitochondrial respiration to support tissue repair and immunoregulatory functions. This metabolic state is closely associated with the activation of transcriptional regulators such as PPARγ and STAT6, as well as increased production of anti-inflammatory cytokines such as IL-10 (55, 56). Importantly, lipid metabolism and mitochondrial fitness are not only energy sources but also key determinants of macrophage functional stability.

Emerging evidence further indicates that additional metabolic pathways, including amino acid metabolism and lactate signalling, contribute to macrophage polarisation in the tumour microenvironment. For example, arginine metabolism regulated by Arg1 can suppress T-cell activation (57), whereas tumour-derived lactate may promote immunosuppressive and pro-angiogenic macrophage phenotypes through epigenetic and transcriptional mechanisms (58). Hypoxia-driven metabolic adaptation also plays a critical role by reshaping both glycolytic and mitochondrial pathways, thereby linking oxygen availability to macrophage functional outputs (59).

Taken together, these findings suggest that metabolic reprogramming operates as an integral component of the hierarchical regulatory network governing macrophage polarisation, acting in concert with cytokine signalling and transcriptional control to determine macrophage functional states in osteosarcoma.

In addition to metabolic regulation, intercellular communication within the tumour microenvironment further refines macrophage functional states, among which exosome-mediated signalling has emerged as an important mechanism. Exosomes play an important role in intercellular communication and can originate from mesenchymal stem cells (MSCs), tumour cells and immune cells. They influence the polarisation process of macrophages by delivering specific molecules, such as microRNAs (miRNAs) or proteins (60). However, exosome-mediated regulation is not a passive process of molecular transfer, but involves selective cargo loading and targeted uptake mechanisms. Cargo selection into exosomes is thought to be regulated by multiple processes, including ESCRT-dependent pathways, RNA-binding proteins, and lipid raft-associated sorting mechanisms, which enable the preferential enrichment of specific miRNAs, proteins, and metabolites under defined cellular conditions (61).

In addition, the uptake of exosomes by recipient cells exhibits a degree of specificity, which may be mediated by surface molecules such as integrins, tetraspanins, and heparan sulfate proteoglycans. These interactions can influence cell-type targeting, internalisation pathways, and downstream signalling responses. For example, tumour-derived exosomes carrying immunoregulatory miRNAs may preferentially interact with macrophages and promote M2-like polarisation (62), whereas macrophage-derived exosomes can reciprocally modulate tumour cell behaviour through receptor-mediated uptake and intracellular signalling reprogramming (63).

### 3.4 Hierarchical network dynamics and feedback regulation

Building on the multi-layered regulatory mechanisms described above, macrophage polarisation can be more accurately understood as an emergent property of an integrated and hierarchical signalling network, rather than the outcome of any single pathway acting in isolation. Within this network, pathway dominance is not fixed but emerges from the relative strength, duration, and integration of upstream signals. For example, acute and high-intensity inflammatory stimuli, such as Toll-like receptor activation or IFN-γ exposure, tend to favour NF-κB-driven transcriptional programmes and promote M1-like activation. In contrast, sustained exposure to tumour-derived cytokines, including IL-4, IL-13, IL-10, and CSF-1, can progressively shift signalling dominance toward STAT3/STAT6- and PI3K-AKT-associated pathways, thereby stabilising M2-like phenotypes. AMPK may function as a context-sensitive metabolic regulator within this hierarchy, modulating downstream outputs depending on nutrient availability, cellular energy status, and crosstalk with inflammatory signalling axes.

Importantly, temporal dynamics play a critical role in shaping macrophage polarisation states. Transient activation of pro-inflammatory pathways may induce reversible M1-like responses, whereas chronic or repetitive stimulation can lead to adaptive reprogramming, including tolerance, exhaustion-like phenotypes, or compensatory activation of immunosuppressive circuits. Similarly, prolonged exposure to tumour-associated signals can reinforce M2-like states through positive feedback loops involving cytokine secretion, metabolic rewiring, and epigenetic modifications. These observations indicate that macrophage polarisation is determined not only by signalling composition, but also by signalling duration, sequence, and cumulative exposure, with early-phase activation and late-phase stabilisation governed by partially distinct regulatory mechanisms.

Within this framework, key pathways such as NF-κB, STAT3/STAT6, PI3K-AKT, and AMPK can be viewed as regulatory nodes that are linked through multiple layers of positive and negative feedback. For example, NF-κB-driven inflammatory responses may transiently suppress M2-associated transcriptional programmes, whereas sustained STAT3/STAT6 activation can counteract pro-inflammatory signalling and reinforce immunosuppressive phenotypes. In addition, metabolic regulators such as AMPK and mTOR form feedback loops with inflammatory pathways, allowing cellular energy status to influence transcriptional outputs and stabilise specific activation states.

Importantly, node dominance within this network is governed not only by signal strength but also by signalling thresholds and cooperative interactions. Certain pathways may only exert functional influence once activation exceeds a critical threshold, beyond which downstream transcriptional and metabolic programmes become self-reinforcing. This threshold-dependent behaviour may explain why macrophage phenotypes can remain stable under fluctuating conditions, yet switch rapidly once cumulative signals surpass a tipping point. Furthermore, feedback amplification, including cytokine autocrine loops (e.g., IL-10, TGF-β) and metabolic reinforcement mechanisms, can lock macrophages into specific functional states, thereby contributing to the persistence of M2-like phenotypes in the tumour microenvironment.

Taken together, this systems-level perspective suggests that macrophage polarisation is best understood as a dynamic network process characterised by node competition, feedback regulation, and threshold-dependent state transitions. This framework not only helps reconcile apparently conflicting findings across studies but also provides a more rational basis for therapeutic intervention, as effective strategies may require simultaneous modulation of multiple regulatory nodes rather than targeting a single pathway in isolation.

### 3.5 Single-cell and spatial perspectives on TAM heterogeneity

Recent advances in single-cell RNA sequencing (scRNA-seq) and spatial transcriptomics have provided critical insights into the functional heterogeneity of tumour-associated macrophages, further challenging the traditional M1/M2 classification framework. These high-resolution approaches have demonstrated that TAM populations are composed of multiple transcriptionally and functionally distinct states rather than discrete subsets (64, 65).

For instance, subsets of TAMs characterised by pro-angiogenic signatures, including high expression of VEGFA, MMPs, and hypoxia-associated genes, have been described as “angiogenic TAMs” (66). Similarly, macrophages enriched for lipid metabolism, oxidative phosphorylation, and mitochondrial gene programmes may be defined as “metabolic TAMs” (67), whereas those expressing immunosuppressive mediators such as IL-10, TGF-β, PD-L1, and ARG1 are often referred to as “immunoregulatory TAMs” (68).

Importantly, these functional states are not mutually exclusive. Individual macrophages may simultaneously exhibit features of multiple programmes, consistent with a continuum model of activation rather than a binary classification.

In addition, spatial transcriptomic analyses have revealed that TAM subsets exhibit non-random spatial organisation within the tumour microenvironment. Angiogenic and immunoregulatory TAMs are often enriched in hypoxic tumour cores or perivascular niches, whereas macrophages with more inflammatory or antigen-presenting features may be relatively more abundant at the invasive margin (69, 70).

Taken together, these findings suggest that macrophage polarisation should be interpreted as a multidimensional and spatially organised process integrating transcriptional, metabolic, and microenvironmental inputs. Incorporating this perspective provides a more accurate framework for understanding TAM function in osteosarcoma and may facilitate the identification of more precise therapeutic targets and biomarker strategies.

## 4 Macrophage polarisation and osteosarcoma progression

**Figure 2:**
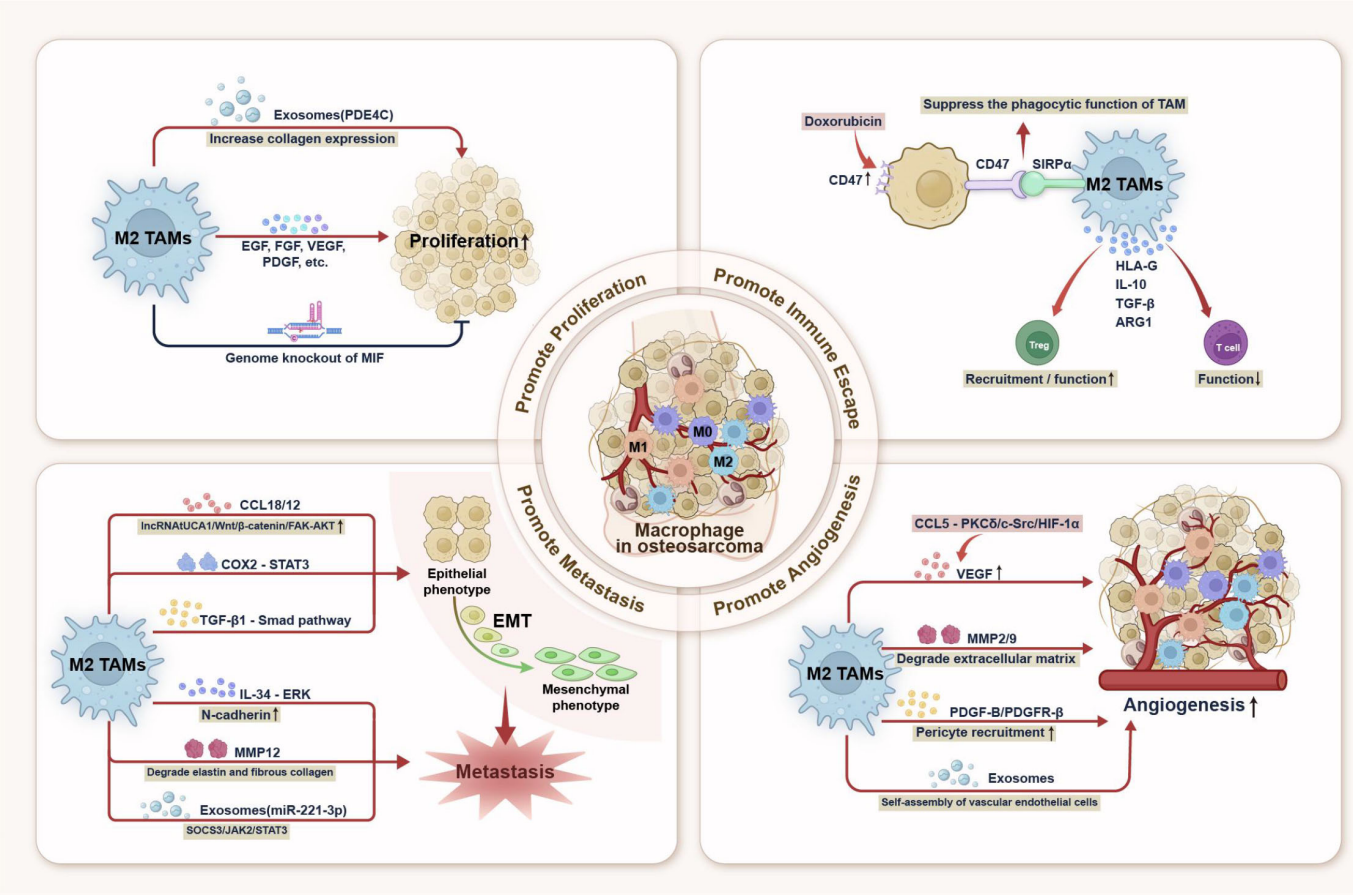
Schematic diagram of macrophage polarisation promoting osteosarcoma progression.

TAM interacts with osteosarcoma cells via autocrine and paracrine signalling, thereby promoting disease progression jointly (71). As illustrated in **Figure 2**, TAM polarisation involves a complex regulatory network in which M1-like and M2-like states represent extremes of a continuous phenotypic spectrum rather than discrete subsets. M2-like TAMs are generally associated with protumour functions such as promoting proliferation, immune suppression, metastasis, and angiogenesis (72–74) (**Figure 2**), these functional associations should not be interpreted as evidence of discrete macrophage subsets. Instead, TAM populations involved in these processes are more likely to represent overlapping or transitional activation states within a continuous phenotypic spectrum, often characterised by the co-expression of both pro-inflammatory and immunoregulatory markers.

Importantly, osteosarcoma progression should not be understood as the result of isolated macrophage–tumour cell interactions, but rather as the outcome of a highly interconnected tumour microenvironmental network. Within this ecosystem, multiple stromal and immune cell populations—including cancer-associated fibroblasts (CAFs), T-cell subsets, neutrophils, and endothelial cells—dynamically interact to collectively shape tumour behaviour. Rather, TAMs function as key but not solitary regulators within this network, T-cell subsets, neutrophils, endothelial cells and extracellular matrix components, all of which can actively shape macrophage polarisation and determine whether TAM-targeted interventions succeed or fail.

Importantly, these interactions are not unidirectional. While tumour cells and stromal components can influence macrophage polarisation, TAMs can in turn modulate fibroblast activation states, regulate T-cell recruitment and effector function, and influence neutrophil behaviour, thereby contributing to reciprocal and multi-layered regulatory loops within the tumour microenvironment.

Mechanistically, TAMs play a central role in shaping T-cell dysfunction within the tumour microenvironment. For example, TAMs can promote T-cell exhaustion through the expression of immune checkpoint ligands such as PD-L1, as well as through the secretion of immunosuppressive cytokines including IL-10 and TGF-β, which impair T-cell proliferation and effector function (75, 76). In addition, TAMs can interfere with effective antigen presentation by downregulating major histocompatibility complex (MHC) molecules and co-stimulatory signals, thereby limiting T-cell activation and contributing to immune evasion (77). Furthermore, TAMs are closely linked to the recruitment and functional regulation of myeloid-derived suppressor cells (MDSCs), which further amplify immunosuppressive signalling within the tumour microenvironment (78, 79). These interactions create a coordinated immunosuppressive network in which macrophages, MDSCs, and dysfunctional T cells act in concert to inhibit anti-tumour immunity.

Importantly, the functional contribution of TAMs is also influenced by their spatial distribution and temporal evolution within the tumour microenvironment. For example, macrophages located in hypoxic tumour cores are more likely to exhibit immunosuppressive and pro-angiogenic characteristics, whereas those at the invasive margin may retain partial inflammatory activity or participate in tissue remodelling and immune cell recruitment (80). In addition, these states may dynamically shift during tumour progression or in response to therapeutic interventions, further underscoring the need to interpret TAM function within a spatiotemporal and continuum-based framework.

Taken together, these observations indicate that TAMs should not be viewed as central drivers in isolation, but rather as integral components of a dynamic and cooperative cellular ecosystem. The functional impact of macrophages therefore depends not only on their intrinsic activation states, but also on their interactions with surrounding stromal and immune populations, as well as the broader spatial and molecular context of the tumour microenvironment.

### 4.1 Promoting the growth and proliferation of osteosarcoma

Osteosarcoma cells induce M2 polarisation by secreting cytokines such as IL-34. Meanwhile, M2-like TAMs accelerate osteosarcoma growth through various mechanisms, creating a malignant cycle (74, 81). These mechanisms primarily involve the secretion of exosomes and the synthesis and secretion of various growth factors and cytokines. Importantly, this tumour-promoting loop may be further reinforced by other stromal constituents within the TME. For example, fibroblast-derived cytokines and matrix-remodelling signals may enhance macrophage recruitment, sustain M2-skewing cues and amplify the proliferative support that TAMs provide to osteosarcoma cells (82).

As a member of the phosphodiesterase 4 (PDE4) family, PDE4C hydrolyses the intracellular second messenger cAMP. This downregulates the cAMP-PKA signalling pathway, thereby participating in the regulation of various processes, such as inflammation, immunity and cellular behaviour. Research indicates that PDE4C, which is derived from M2-like TAM exosomes, may enhance the proliferative capacity of osteosarcoma cells by increasing collagen expression (83). Furthermore, it has been widely confirmed that the AKT signalling pathway is one of the key molecular pathways driving tumour progression. Exosomes released by M2-like TAMs can promote tumour cell proliferation by activating this pathway (84).

The M2 type of TAM can secrete various growth factors, including epidermal growth factor (EGF), fibroblast growth factor (FGF), vascular endothelial growth factor (VEGF) and platelet-derived growth factor (PDGF). These factors stimulate the direct proliferation of osteosarcoma cells and enhance their resistance to apoptotic stimuli (85). Macrophage migration inhibitory factor (MIF) is another multifunctional cytokine that is secreted by M2-like TAMs and promotes tumour progression in the tumour microenvironment by enhancing proliferation, inhibiting apoptosis and inducing angiogenesis (86). Han et al. found that knocking down the MIF gene can inhibit osteosarcoma proliferation, further indicating that MIF derived from M2-like TAMs plays a crucial role in osteosarcoma growth promotion (87). Taken together, these findings suggest that TAM-mediated growth promotion is not merely a direct effect on tumour cells, but part of a multi-cellular trophic network in which soluble mediators, exosomes and stromal remodelling signals cooperate to sustain osteosarcoma expansion.

### 4.2 Promoting immune evasion of osteosarcoma

The body combats tumours through innate and adaptive immune surveillance. Impairment of this function can result in the uncontrolled proliferation of tumour cells (88). Although chemotherapy can activate the immune system to some extent, enabling it to eliminate tumour cells, studies have shown that tumours may become more resistant to the immune system after chemotherapy (89). Analysis of osteosarcoma specimens before and after chemotherapy indicates that metastatic patients have significantly higher numbers of M2-like TAMs than non-metastatic patients (72). This suggests that, during chemotherapy, M2-like TAMs may contribute to the development of an immunosuppressive microenvironment, thereby facilitating immune evasion via specific mechanisms. These mechanisms include inhibiting macrophage phagocytosis, suppressing T cell function and recruiting suppressive/regulatory T cells (Tregs). Notably, this immunosuppressive phenotype is not generated by TAMs alone, but emerges from coordinated interactions between TAMs and other immune and stromal elements in the TME, particularly exhausted CD8^+^ T cells, Tregs and metabolically restrictive extracellular conditions (90).

CD47 is a transmembrane glycoprotein which is highly expressed in osteosarcoma cells, and which is closely associated with a poor prognosis for patients. In the TME of osteosarcoma, CD47 binds to signal regulatory protein alpha (SIRPα) on the surface of TAMs, transmitting a ‘don’t eat me’ signal that inhibits phagocytosis by macrophages, thereby helping tumour cells to evade immune attack (91). Notably, chemotherapeutic agents such as doxorubicin can prompt tumour cells to release specific factors that increase CD47 expression, thereby enhancing tumour immune evasion post-chemotherapy (92).

The M2 TAM subtype can also maintain a sustained immunosuppressive state in the TME by secreting mediators such as HLA-G, IL-10 and transforming growth factor beta (TGF-β). These molecules directly inhibit the functions of effector T cells and recruit Tregs, thereby enhancing immune evasion further (93). In terms of specific mechanisms, TGF-β plays a dual role. On the one hand, it inhibits the proliferation of CD8^+^ T cells directly, obstructs the release of cytotoxic granules such as perforin and granzyme, and downregulates the expression of IL-2 receptors and IFN-γ. This blocks the cytotoxic function of T cells (94). On the other hand, TGF-β can work together with chemokines, such as CCL22, to attract Tregs to the tumour microenvironment. Through activation of the Foxp3 signalling pathway, TGF-β induces the differentiation of naïve CD4+ T cells into Tregs, which continuously suppress the local immune response (84). Furthermore, high concentrations of IL-10 can prevent dendritic cells from secreting IL-12, which weakens the ability of CD8^+^ T cells to recognise osteosarcoma cells (95). HLA-G has been linked to the functional inhibition of CD8^+^ T cells and natural killer (NK) cells, and it is thought to promote immune evasion by affecting the cytotoxic efficacy of chimeric antigen receptor (CAR) T cells (96). Notably, M2-like TAMs express high levels of arginase-1 (Arg1), which depletes arginine in the TME and inhibits the activation and proliferation of effector T cells. Furthermore, activating metabolic stress signals, such as GCN2, enhances the immunosuppressive function of M2-like TAMs, constituting a key metabolic mechanism of immune evasion in osteosarcoma (97). This metabolic mechanism is particularly important because it suggests that TAM-mediated arginine depletion may act synergistically with classical immune checkpoint pathways, jointly reinforcing CD8^+^ T-cell dysfunction and exhaustion rather than operating as an independent suppressive process. In this sense, the immune landscape of osteosarcoma is shaped by layered inhibitory circuits, in which cytokine-mediated suppression, metabolic deprivation and checkpoint signalling converge to weaken antitumour immunity.

Moreover, the spatial distribution and temporal evolution of TAM states may critically influence these functional outcomes, suggesting that the impact of macrophages on osteosarcoma progression cannot be fully understood without considering their dynamic positioning and state transitions within the tumour microenvironment.

### 4.3 Promoting metastasis of osteosarcoma

The primary reason for the low five-year survival rate in patients with osteosarcoma is tumour metastasis, with the lungs being the most common site of metastasis, accounting for up to 83.8% of cases (98). The proportion of M2-like TAMs in metastatic osteosarcoma tissue is significantly higher than in localised cases. This is associated with an increased ability of co-cultured osteosarcoma cells to form colonies, suggesting a close relationship between M2 polarisation and metastasis (83). The currently known mechanisms by which M2-like TAMs promote metastasis include cytokine secretion, intercellular communication via exosomes and protease activity regulation. However, metastatic dissemination is not driven by TAMs alone. Rather, it reflects cooperative remodelling of the metastatic niche by macrophages together with fibroblastic stromal cells, extracellular matrix components and other myeloid populations such as neutrophils.

Epithelial-mesenchymal transition (EMT) is the process by which epithelial cells transform into a mesenchymal phenotype. This process is crucial for tumour cells to acquire invasive and migratory capabilities. In osteosarcoma, M2-like TAMs can promote the EMT process by secreting various cytokines and mediators, thereby enhancing the tumour’s metastatic potential. Specifically, CCL18 and CCL22, which are derived from M2-like TAM, drive the expression of EMT-related genes and accelerate osteosarcoma metastasis by upregulating the long non-coding RNA (lncRNA) UCA1/Wnt/β-catenin and focal adhesion kinase (FAK)-AKT signalling pathways (99–101). Additionally, TAM of the M2 type can upregulate the expression of cyclooxygenase-2 (COX-2) in osteosarcoma cells. This induces EMT through activation of the COX-2/STAT3 axis, thereby promoting tumour invasion and lung metastasis (72). Notably, TGF-β1 can activate EMT-related transcription programmes via the Smad-dependent pathway, while simultaneously inhibiting the BMP antagonistic pathway. Furthermore, it can reduce the expression of Smad2/3-mediated miR-143, which exacerbates the migration and invasion of osteosarcoma cells (102). Moreover, IL-34 increases the expression of N-cadherin by activating the ERK signalling pathway, thereby promoting osteosarcoma metastasis (103). In addition to EMT regulation, remodelling of the extracellular matrix is another important mechanism by which M2-like TAMs promote metastasis. The secretion of matrix metalloproteinase 12 (MMP-12) can degrade elastin and fibrillar collagen efficiently, thereby compromising vascular stability and creating conditions that allow tumour cells to spread to other parts of the body (104). Importantly, extracellular matrix remodelling should be viewed as a multi-compartment process rather than a macrophage-restricted event. CAFs and matrix-associated stromal programmes can reinforce TAM-derived proteolytic activity, increase tissue stiffness and alter migratory tracks for tumour cells, thereby amplifying metastatic competence.

In addition, as one of the important communication mechanisms in the tumour microenvironment, exosomes participate in the regulation of osteosarcoma metastasis by delivering functional molecules. For example, Liu et al. showed that introducing miR-221-3p mimics into M2-like TAMs greatly increases the migratory and invasive abilities of human osteosarcoma cells via the exosomes released by these macrophages. Further mechanistic analysis revealed that this effect is mediated by exosomal miR-221-3p targeting the SOCS3/JAK2/STAT3 signalling axis (105). Neutrophils may also contribute to this prometastatic ecosystem by supplying inflammatory mediators, proteases and extracellular traps that facilitate tumour cell survival and dissemination, suggesting that TAM-induced metastasis is likely reinforced by broader myeloid crosstalk within the osteosarcoma microenvironment.

### 4.4 Promoting angiogenesis in osteosarcoma

Angiogenesis is a critical process that supplies tumours with oxygen and nutrients, playing a vital role in the progression of osteosarcoma (106). M2-like TAMs are one of the main drivers of angiogenesis (107). Their pro-angiogenic mechanisms include activating key signalling pathways, secreting pro-angiogenic factors and recruiting pericytes, to name a few. Yet the angiogenic programme of osteosarcoma is not solely macrophage-driven. Instead, it emerges from reciprocal interactions among TAMs, endothelial cells, pericytes, fibroblastic stromal cells and extracellular matrix components, which collectively regulate vessel sprouting, maturation and stability (3).

Early studies have found a positive correlation between the degree of TAM infiltration in tumour tissues and vascular density (108). VEGF is a core factor that promotes tumour angiogenesis by M2-like TAM. It can be upregulated through the CCL5-mediated PKCδ/c-Src/HIF-1α pathway, thereby enhancing the VEGF-dependent angiogenesis process (109). Furthermore, the high expression of matrix metalloproteinases (MMPs), such as MMP-2 and MMP-9, by M2-like TAMs can degrade the extracellular matrix and release additional pro-angiogenic factors. This promotes endothelial cell migration and tubular structure formation, thereby accelerating angiogenesis in osteosarcoma (110).

Platelet-derived growth factor B (PDGF-B) is a member of the PDGF family. M2-like TAMs express high levels of PDGF-B, which, via the PDGF-B/PDGFR-β axis, recruits pericytes to the periphery of newly formed blood vessels, thereby enhancing the stability and integrity of the vasculature (111). Meanwhile, the release of exosomes by M2-like TAMs can induce endothelial cells to self-assemble into capillary-like structures by delivering specific mRNA and other substances. This process continuously promotes angiogenesis in osteosarcoma (112, 113). Because fibroblasts and matrix components also influence endothelial behaviour and growth factor availability, the angiogenic consequence of TAM activity is likely to depend on the broader stromal context. This may help explain why antiangiogenic or TAM-modulating strategies show variable efficacy across models and suggests that successful vascular remodelling will require consideration of the entire microenvironmental ecosystem rather than macrophages alone.

Overall, TAMs should be regarded as central coordinators of osteosarcoma progression, but not as autonomous effectors. Their functions are continuously shaped by reciprocal communication with CAFs, T-cell subsets, neutrophils, endothelial cells and extracellular matrix components. This broader network perspective is particularly important for understanding therapeutic resistance: a TAM-targeted intervention may produce limited benefit if parallel stromal or immune suppressive circuits remain intact, whereas combined disruption of macrophage signalling together with T-cell exhaustion pathways, fibroblast-derived cues or matrix remodelling programmes may achieve more durable antitumour effects. Accordingly, mechanistic interpretation of TAM biology in osteosarcoma should move from a cell-centric model towards an ecosystem-based framework.

## 5 Strategies for modulating macrophage polarisation to inhibit osteosarcoma

Although there has been some progress in the treatment of osteosarcoma in recent decades, the overall prognosis remains poor (114). Therefore, finding more effective treatment methods has become a critical issue that needs to be addressed in clinical practice. Immunotherapy, including immune checkpoint inhibitors, is an emerging therapeutic direction in the field of oncology that has shown encouraging activity in preclinical models, although clinical benefit remains to be established (115). However, despite these advances, clinical trial data specifically evaluating TAM-targeted strategies in osteosarcoma remain limited, and most current evidence is derived from early-phase studies or extrapolated from other tumour types. Of these, strategies targeting the polarisation of macrophages have significant potential for application due to their unique advantages. Macrophages exhibit phenotypic plasticity, enabling them to switch between the antitumour M1 and protumour M2 phenotypes (116). Another clinical study has confirmed that the polarisation state of macrophages may be related to the efficacy of chemotherapy and the prognosis of patients with osteosarcoma (117).

However, the therapeutic approaches discussed below should not be regarded as having equivalent translational readiness. Conventional small molecules, repurposed agents and clinically used immunomodulators are generally supported by more established pharmacological and safety data, whereas advanced platforms such as cytokine recycling systems, biomimetic nanoparticles, sonodynamic therapy and photothermal therapy remain largely at an early preclinical stage. Accordingly, their striking efficacy in cell culture and murine models should be interpreted with caution, because major translational barriers remain unresolved, including tumour penetration, payload stability, biodistribution, large-scale manufacturing, on-target or off-tumour toxicity, and the limited ability of current mouse models to recapitulate the complexity of human osteosarcoma immunobiology.

Based on current research findings, strategies for inhibiting osteosarcoma by modulating macrophage polarisation can be categorised into three main approaches. The first approach involves repolarising M2 macrophages back to the M1 phenotype to reverse their pro-cancer functions. The second approach involves inducing the polarisation of TAM towards the M1 phenotype to enhance their anti-tumour activity. The third approach involves inhibiting the transition of macrophages from the M1 phenotype to the M2 phenotype to maintain an anti-tumour immune environment within the body (**Table 1**). Importantly, this classification is intended as a mechanistic framework rather than an implication of comparable clinical feasibility. From a translational perspective, these strategies differ substantially in their technology readiness levels, manufacturability and regulatory complexity.

**Table 1:** Strategies targeting TAM polarisation for osteosarcoma treatment.

Regulatory strategy	Intervention method	Representative drug	Ref.
Repolarising M2 TAM towards M1 TAM	CSF-1R inhibitor	Pexidartinib	(119)
	Targeting key regulatory genes	*BNIP3* knockdown	(120)
	Immune checkpoint inhibitors/combination therapy	Anti-PD1 treatment	(124)
		Anti-CD47 mAb combined with doxorubicin	(125)
	Natural products	β-caryophyllene	(128–130)
		Chalcone compounds (Xanthoangelol or 4-hydroxyderricin)	(131)
		Dihydroxycoumarins (esculetin or fraxetin)	(133)
		Asiaticoside (ATS)	(132)
	Drug repurposing	All-Trans retinoic acid (ATRA)	(51)
		Metformin	(134, 135)
	Nanomaterials	Mn-ZIF nanozymes	(138)
		Ruthenium Nanoreactors (NP4)	(139)
	Sonodynamic therapy (SDT)	MPIRx biomimetic nanodrug	(142)
Inducing the original TAM polarisation towards M1 TAM	Cytokine efficacy recovery system (CERS)	IFN-γ cmRNA@CERS or combined with the PD-L1 mAb	(146)
	Targeting Toll-like receptor 4 (TLR4)	Zoledronic acid (ZA)	(150, 151)
		Liposomal muramyl tripeptide (MTP-PE) or combined with ZA	(152, 153)
	TrK inhibitor	Larotrectinib	(81)
	Natural products	Arjunolic acid (AA)	(157)
	Biomimetic cell membrane nanoparticles	Signal regulatory protein alpha (SIRPα)-membrane@nano-Prussian blue (SIRPα-M@nanoPB)	(91, 158, 159)
	Photothermal therapy (PTT)	Graphene oxide (GO)-polyethylene glycol (PEG) photothermal material (GO-PEG)	(161)
Inhibiting M1-to-M2 TAM polarisation	Targeting chemokines	CCL2 inhibitor (Bindarit)	(166)
	Targeting transcription factors	Overexpression of paired-like homeodomain transcription factor 1 (PITX1)	(100)

### 5.1 Repolarising M2-like TAM to the M1 form

As previously mentioned, various cytokines, including IL-34 and CSF-1, can induce type 2 polarisation in TAMs. Therefore, targeting M-CSF/IL-34 is an important strategy for repolarising M2 to M1. Kubota et al. confirmed that M-CSF inhibitors selectively suppress tumour angiogenesis and lymphangiogenesis in osteosarcoma mice, reducing tumour regrowth after treatment cessation (118). Building on this, Fujiwara et al. demonstrated through *in vivo* and *in vitro* experiments that the CSF-1R inhibitor PLX3397 (pexidartinib) reduces TAM infiltration and migration, and promotes the transition from M2 to M1. This has been shown to inhibit osteosarcoma growth and metastasis in preclinical models (119). BNIP3 (BCL2-interacting protein 3) is a key gene associated with TAM polarisation and poor prognosis in osteosarcoma. It exhibits high expression in osteosarcoma cell lines (120). Knockdown of BNIP3 also promotes the repolarisation of M2 to M1, as demonstrated by co-culture experiments showing a restoration of M1 proportions and CD86 expression (120). In addition, the inhibition or knockdown of other targets, such as lysyl oxidase (LOX), the macrophage receptor with collagenous structure (MARCO) and high-mobility group box 1 (HMGB1), has also demonstrated the potential to induce M2 reprogramming (121–123). Further investigation into the underlying mechanisms and clinical trials are warranted. From a translational standpoint, agents directed against CSF-1 or CSF-1R are relatively more advanced than most experimental platforms because they target a pharmacologically tractable axis and benefit from broader oncological experience. Nevertheless, their clinical application in osteosarcoma still faces important challenges, including incomplete macrophage reprogramming, compensatory immunosuppressive circuits and the risk of systemic effects on physiological macrophage populations in normal tissues.

Anti-PD-1 therapy has been shown to be capable of modulating macrophage phenotypes and producing antitumour effects in preclinical osteosarcoma models. Studies have demonstrated that anti-PD-1 treatment is associated with a notable increase in M1-like TAMs and a significant decrease in M2-like TAMs compared to the control group. However, TAM depletion significantly reduces the efficacy of anti-PD-1 therapy (124). This suggests that the conversion of M2-like TAMs into M1-like TAMs is a key mediator of the therapeutic effect of anti-PD-1. These findings provide a mechanistic rationale for combining anti-PD-1 therapy with other immunotherapies to treat osteosarcoma patients. For example, Mohanty et al. discovered that, compared to the use of anti-CD47 monoclonal antibodies or doxorubicin in isolation, a combination of the two enhances the phagocytic activity of M1-like TAMs against osteosarcoma cells and significantly reduces M2-like TAM markers. This demonstrates a strong additive effect in osteosarcoma models (125). Nevertheless, the encouraging preclinical activity of checkpoint blockade in osteosarcoma should be interpreted conservatively. Clinical responses to immune checkpoint inhibitors in osteosarcoma have generally been less impressive than those observed in more immunogenic malignancies, suggesting that macrophage reprogramming alone may be insufficient unless accompanied by broader remodelling of the tumour immune microenvironment.

Products derived from medicinal plants or dietary foods also exhibit anti-tumour and cancer-preventive properties (126). In recent years, β-caryophyllene, a naturally occurring bicyclic sesquiterpene, has attracted considerable attention for its potential anticancer activity (127). Zhang et al.’s study found that β-caryophyllene can inhibit the tumour-promoting ability of osteosarcoma stem cells *in vivo* and reduce the tumour-promoting characteristics of doxorubicin-resistant cells. This suggests a potential to enhance the efficacy of osteosarcoma chemotherapy in preclinical settings. Differential analysis revealed a substantial rise in the quantity of M1-like TAMs. Immunohistochemical findings revealed that, compared to the control group, the β-caryophyllene group exhibited notably higher levels of M1 macrophage markers iNOS and CD86, and notably lower levels of M2 macrophage markers Arg1 and CD206 (128). The underlying mechanism may involve β-caryophyllene promoting the polarisation of M1 macrophages and the secretion of inflammatory cytokines through the p53-AKT-FoxO1 signalling pathway (129, 130). In addition, yellow angelica alcohol, extracted from Angelica sinensis, and natural coumarin derivatives, such as 4-hydroxyderricin, esculetin and fraxetin, as well as the Asiaticoside (ATS), derived from Centella asiatica, have all been reported to have anti-osteosarcoma effects. This is achieved by pulling TAMs that have already polarised, or are currently polarising, towards the M2 phenotype back to the M1 phenotype. This process involves blocking M2-like TAM activation and differentiation (131–133). These compounds are attractive because of their relative accessibility and conceptual simplicity; however, their translational advancement remains limited by incomplete pharmacokinetic characterisation, uncertain bioavailability at tumour sites, batch-to-batch variability for natural products and the frequent lack of rigorous toxicity and dose-optimisation studies in clinically relevant models.

It has also been shown that all-trans retinoic acid (ATRA) and metformin can induce the repolarisation of M2 to M1. ATRA can reduce the number of M2-like TAMs in osteosarcoma and inhibit lung metastasis (51). Metformin can induce the transition from M2 to M1, thereby decreasing the number of myeloid-derived suppressor cells (MDSCs) and leading to anti-tumour and anti-angiogenic effects (134, 135). Compared with many emerging platforms, drug repurposing strategies such as ATRA and metformin may possess a comparatively higher translational feasibility because their pharmacology and systemic safety profiles are already partially defined in humans. Even so, whether the doses required to remodel TAM phenotypes in osteosarcoma are clinically achievable, and whether such effects can be sustained without off-target metabolic consequences, remain open questions.

Thanks to advances in nanotechnology, nanomaterials that are biocompatible, modifiable and capable of loading high amounts of drugs have become important tools for regulating the TME. Manganese-containing nanoparticles, for example, can activate the NF-κB signalling pathway by generating reactive oxygen species (ROS). This promotes the transformation of TAM into a pro-inflammatory phenotype, thereby enhancing their cytotoxicity against osteosarcoma cells in experimental systems (136, 137). Han et al.’s Mn-ZIF nanoenzyme was found to significantly inhibit osteosarcoma cell proliferation and reduce cell volume *in vitro* at a concentration of 200 μg/mL (138). Subsequently, its ability to reprogram the immune microenvironment was confirmed in a subcutaneous osteosarcoma model in nude mice. Compared to the control group and the single ZIF group, the proportion of M1-like TAM in the tumour region increased more than fivefold in the Mn-ZIF group, while the proportion of M2-like TAM decreased significantly, suggesting that this nanoenzyme may reverse the TAM polarisation state under experimental conditions. Given its good biosafety profile, Mn-ZIF shows promise as an immunoadjuvant therapy for osteosarcoma. Although these results are encouraging, the translational readiness of such nanoplatforms remains low. Apparent biosafety in short-term mouse studies does not adequately predict human safety, particularly with respect to long-term retention, hepatic or splenic accumulation, immunogenicity and unintended inflammatory injury in non-tumour tissues. In addition, efficient penetration into dense osteosarcoma lesions and metastatic niches, especially in the lung, remains a substantial delivery challenge for nanoparticle-based systems. Additionally, the tumour-targeting ‘nano-reactor’ NP4, which is self-assembled from ruthenium catalysts and perfluorocarbon side-chain polymers, can increase the number of M1-like TAMs and decrease the number of M2-like TAMs in osteosarcoma tissue. This has been shown to inhibit tumour growth *in situ* in preclinical models (139). However, the complexity of nano-reactor design may itself become a translational obstacle, because sophisticated multi-component systems often face difficulties in scalable manufacturing, reproducibility, regulatory evaluation and quality control.

Sonodynamic therapy (SDT) is a treatment strategy that uses low-intensity ultrasound to activate sonosensitizers and generate ROS through ultrasound cavitation, creating a localised oxidative stress microenvironment. This microenvironment directly induces apoptosis in tumour cells and promotes M1 polarisation (140, 141). Gong et al. developed MPIRx nanoparticles that co-load the sonosensitiser IR780 and the CD47 inhibitor RRx-001 (142). The study confirmed that, following ultrasound activation, MPIRx can effectively convert M2-like TAMs into M1-like cells, which has been shown to inhibit osteosarcoma growth in preclinical models and reduces the risk of lung metastasis. Furthermore, SDT has good tissue penetration capabilities, making it a promising candidate for combined therapy in the treatment of osteosarcoma, involving both cell killing and immune modulation of macrophages (142). SDT is conceptually appealing because it combines physical targeting with immune modulation; however, its clinical translation remains preliminary. Its efficacy depends on the precise coordination of sonosensitizer accumulation, acoustic energy delivery and tumour accessibility, all of which may vary substantially between subcutaneous mouse models and deeply situated human osteosarcoma lesions. Moreover, off-target oxidative damage, tissue heating and heterogeneity in ultrasound penetration should be carefully considered before extrapolating preclinical efficacy to clinical settings.

### 5.2 Inducing the polarisation of primitive TAM towards the M1-like TAM

By providing M1-polarised signals, the original TAM can be guided directly to develop an anti-tumour phenotype. Among these signals, IFN-γ is a key cytokine that induces M1 polarisation (143). The combination of LPS and IFN-γ activates M1-like TAM, which can release factors such as TNF-α and IL-1β that significantly inhibit the growth of osteosarcoma cells (144, 145).

Drawing inspiration from the Kinetic Energy Recovery System (KERS), Shao et al. designed a CERS (146). This system can recover around 40% of cytokines by capturing them in a mineral coating, which extends their action time. By delivering IFN-γ mRNA, CERS promotes the polarisation of TAM towards the M1 phenotype, thereby activating adaptive immunity against osteosarcoma. It is worth noting that previous studies have shown that anti-PD-1 treatment can significantly increase the proportion of M1-like TAM within pulmonary metastatic lesions of osteosarcoma. Based on this, combining CERS-mediated IFN-γ mRNA delivery with anti-PD-1 therapy has been reported to induce regression in murine models of highly malignant pulmonary metastatic lesions of osteosarcoma. This is potentially due to both approaches synergistically inducing a robust systemic immune response (146). Despite these remarkable preclinical findings, CERS should be regarded as an early-stage therapeutic concept rather than a near-clinical technology. Its translation will depend on whether mRNA cargo can maintain sufficient stability during delivery, whether cytokine recycling can be controlled without excessive immune activation, and whether the platform can be manufactured reproducibly at scale. In addition, therapeutic success in murine pulmonary metastasis models may overestimate efficacy in patients, in whom immune heterogeneity, prior treatment exposure and variable metastatic architecture are much more complex.

Toll-like receptor 4 (TLR4) is an important pattern recognition receptor involved in innate immunity (147). Studies have found that osteosarcoma tissues have a significantly lower positive expression rate of TLR4 than the surrounding normal tissues. This suggests that the active state of TLR4 may be related to tumour immune regulation (148). Activation of TLR4 signalling has been shown to promote the polarisation of TAM towards the pro-inflammatory M1 phenotype and inhibit the M2 phenotype (149). The commonly used drug zoledronic acid (ZA) activates M1 macrophages via a TLR4-dependent mechanism and inhibits M2 polarisation through the Wnt/β-catenin pathway. This induces apoptosis in osteosarcoma cells, thereby preventing metastasis and angiogenesis (150, 151). Additionally, the liposome-encapsulated innate immune activator L-MTP-PE activates both the TLR4 and NOD2 pathways simultaneously, thereby enhancing M1 polarisation and the release of pro-inflammatory factors synergistically (152). Notably, combining L-MTP-PE and ZA can further enhance the M1 polarisation effect synergistically, thereby improving anti-tumour activity (153). Compared with highly engineered nanoplatforms, TLR4-directed immunomodulatory strategies may be considered closer to translational application because they build on better-characterised immune agonists or clinically used agents. Nevertheless, systemic activation of innate immune pathways carries an inherent risk of excessive inflammation, and the therapeutic window for achieving intratumoural immune activation without unacceptable collateral toxicity remains to be clearly defined.

In addition to TLR4, other signalling pathways can serve as targets for regulating TAM polarisation. For example, TrkA, which belongs to the tropomyosin receptor kinase (Trk) family, can contribute to tumour progression via nerve growth factor (NGF) signalling (154, 155). Lin et al. discovered that NGF promotes the infiltration of TAMs into the microenvironment, driving their polarisation towards the M2 phenotype. However, the Trk family inhibitor larotrectinib can reverse this process (81). Therefore, larotrectinib has emerged as a potential candidate drug for targeting NGF signalling and interrupting the progression of osteosarcoma. Yet here again, mechanistic promise should not be conflated with immediate clinical applicability, because biomarker-defined patient selection, pathway dependence in osteosarcoma and resistance mechanisms remain insufficiently characterised.

Arjunolic acid (AA) is a small, natural molecule with antioxidant and anti-inflammatory properties (156). In the study by Li et al., applying AA to osteosarcoma cells co-cultured with TAM transfected with Wnt3a inhibited the proliferation of osteosarcoma cells and reduced their migration and invasion rates. Further analysis revealed that TAM treated with AA exhibited increased expression of the M1 marker CD86, as well as pro-inflammatory factors such as IL-6 and TNF-α (157). These results suggest that AA can polarise TAMs in the TME towards the M1 phenotype, suggesting inhibition of malignant behaviour in experimental models of osteosarcoma cells. However, as with other natural compounds, the distance from proof-of-concept efficacy to clinical adoption remains substantial.

Bionic nano-strategies based on cell membranes have also shown promise. This approach utilises macrophage membranes that overexpress SIRPα after M1 polarisation to encapsulate nanoparticles (SIRPα-M@nanoPB). These nanoparticles can then specifically bind to CD47 on the surface of osteosarcoma cells and be internalised by TAM. This induces M1 polarisation within the TME (91, 158, 159). This strategy has been shown to enable targeted effects in preclinical systems of osteosarcoma cells, alleviates immune suppression and reduces the immunogenicity of the materials. This facilitates the conversion of ‘cold tumours’ into ‘hot tumours’. This biomimetic strategy is scientifically elegant, but its translational readiness is still limited. Cell membrane-derived platforms may improve targeting and immune evasion, yet they also introduce substantial practical challenges, including source standardisation, membrane isolation consistency, batch reproducibility, storage stability and regulatory classification. Moreover, although CD47-targeted designs may enhance macrophage-mediated clearance of tumour cells, they also raise concern for on-target effects in normal cells expressing CD47, underscoring the need for careful toxicity evaluation.

Photothermal therapy (PTT) is a strategy that uses near-infrared (NIR) light to irradiate photothermal materials and generate heat for the treatment of tumours (160). Deng et al.’s graphene oxide (GO)-polyethylene glycol (PEG) photothermal material induces M1-like differentiation and suppresses M2-like differentiation following low-temperature photothermal treatment. This has been reported to reduce the invasive and migratory capabilities of osteosarcoma cells (161). Nonetheless, the clinical deployment of photothermal strategies in osteosarcoma may be constrained by limited light penetration, heterogeneous heat distribution in deep tissues and the possibility of collateral damage to adjacent normal structures. Therefore, although these technologies are mechanistically attractive, they remain considerably less mature than repurposed pharmacological agents or better-established immunomodulatory approaches.

### 5.3 Inhibiting the polarisation of M1-like TAM towards M2 form

Strategies aimed at suppressing the conversion of M1-like TAMs to M2-like TAMs have shown significant potential when targeting key regulatory nodes such as chemokines and transcription factors (162). Among these, CCL2 is an important chemokine that can be secreted by tumour cells or TAMs themselves. It is responsible for recruiting macrophages to the TME and primarily drives their polarisation from the M1 to the M2 type (163–165). Research has confirmed that the CCL2-specific inhibitor Bindarit reduces the infiltration of macrophages into osteosarcoma tissue, inhibits tumour growth, and has been shown to block the M1 to M2 polarisation process in preclinical models (166). Because chemokine-directed approaches target a relatively defined signalling axis, they may be more straightforward to translate than complex multifunctional nanoplatforms. Even so, inhibition of a single chemokine pathway may be insufficient in the presence of redundant inflammatory circuits within the osteosarcoma microenvironment.

Paired-like homeodomain transcription factor 1 (PITX1) is a transcription factor that is associated with the proopiomelanocortin (POMC) gene. It is involved in regulating pituitary cell differentiation and hindlimb development (167). PITX1 is down-regulated in various tumour tissues and its expression level correlates positively with patient prognosis, suggesting a potential tumour-suppressing function (168–171). Zhang et al.’s study found that PITX1 can promote the proteasomal degradation of STAT3, thereby inhibiting the expression of the long non-coding RNA LINC00662. LINC00662 is often found in high concentrations in exosomes and can facilitate communication between osteosarcoma cells and TAM, thereby promoting M2 polarisation (100). Experimental results indicate that PITX1 overexpression reduces M2 marker levels (CD163, CD206 and Arg-1), while PITX1 knockdown increases M2 markers and downregulates M1-related factors (IL-1β and iNOS). These results suggest that PITX1 may inhibit osteosarcoma progression both *in vitro* and *in vivo* by suppressing M1 to M2 polarisation (100). However, transcription factor-centred strategies are still predominantly mechanistic and experimental. Their translation into clinically actionable therapies will require the development of reliable delivery systems, validated biomarkers and acceptable safety profiles for gene-level intervention.

Overall, the therapeutic landscape of TAM-targeted intervention in osteosarcoma is heterogeneous rather than equivalent. Small-molecule agents, repurposed drugs and selected immunomodulators may be considered relatively closer to clinical translation, whereas advanced delivery systems such as CERS, biomimetic nanoparticles, sonodynamic platforms and photothermal materials remain at earlier technology readiness levels despite impressive proof-of-concept efficacy. A major reason for this discrepancy is that preclinical success is usually demonstrated in simplified mouse models that do not fully capture the anatomical, immunological and therapeutic complexity of human osteosarcoma. In addition, the absence of validated TAM-associated biomarkers, the historically limited responsiveness of osteosarcoma to immunotherapy, and the substantial manufacturing and regulatory burden associated with advanced therapeutic products all complicate clinical translation. Future studies should therefore move beyond demonstrating antitumour effects in principle and instead prioritise biomarker qualification, patient stratification, pharmacokinetics, biodistribution, long-term safety, manufacturability, regulatory feasibility and rational clinical trial design. Only through such clinically grounded evaluation can macrophage-targeted therapies be realistically advanced from promising concepts to meaningful interventions for patients with osteosarcoma.

### 5.4 Clinical translation challenges and future directions for TAM-targeted therapy

Although TAM-targeted strategies have shown considerable promise in preclinical osteosarcoma models, their clinical application still faces several unresolved barriers that extend beyond proof-of-concept efficacy. A major challenge is the lack of robust and clinically actionable biomarkers for defining macrophage polarisation states in patient samples. At present, commonly used markers such as CD68, CD86, iNOS, CD163, CD206 and Arg1 are helpful for research purposes (157, 172), but they do not always provide a sufficiently stable or standardised framework for clinical decision-making. The M1/M2 classification is widely used in research contexts; however, it cannot be directly applied in clinical diagnosis or treatment evaluation due to the dynamic and heterogeneous nature of macrophage phenotypes. In reality, macrophage phenotypes exist along a continuum, and marker expression may vary according to tissue region, treatment exposure and sampling time point. Therefore, relying solely on the M2/M1 ratio or on single-marker immunohistochemistry may oversimplify the biological state of TAMs in osteosarcoma.

Future studies should prioritise the development of integrated biomarker systems combining multiplex immunohistochemistry, single-cell sequencing, spatial transcriptomics and possibly circulating indicators, so that TAM states can be more reliably linked to prognosis, treatment response and patient stratification.

In this context, a multi-parameter biomarker panel may represent a more practical and informative approach than reliance on single markers. Such a panel could include four main components. First, lineage and abundance markers such as CD68 and CD14 can be used to quantify macrophage infiltration. Second, functional polarisation markers reflecting pro-inflammatory and immunoregulatory programmes, including CD86 and iNOS for M1-like features and CD163, CD206 and ARG1 for M2-like features, can provide insight into macrophage activation states. Third, immune checkpoint and immunosuppressive molecules such as PD-L1, IL-10 and TGF-β can be used to assess macrophage-mediated T-cell dysfunction. Fourth, metabolic and microenvironment-associated markers including HIF-1α, GLUT1 and lipid metabolism-related proteins can capture metabolic reprogramming and hypoxia-driven adaptation.

Importantly, the spatial distribution of these markers, for example their localisation within tumour cores, invasive margins or perivascular niches, should be considered alongside their expression levels, as spatial context may critically influence macrophage function and therapeutic response. In addition, integrating these tissue-based markers with circulating indicators such as cytokine profiles or exosome-associated signatures may further enhance the clinical utility of TAM-related biomarker systems.

A second key issue is that immunotherapy has historically shown limited benefit in osteosarcoma compared with more immunogenic malignancies. This likely reflects several factors, including relatively low immunogenicity, a highly suppressive tumour microenvironment, marked inter- and intratumoural heterogeneity, and the complex effects of prior chemotherapy on immune composition and function. In addition, osteosarcoma lesions often contain abundant suppressive myeloid cells, dysfunctional or exhausted T cells, and stromal barriers that may restrict effective immune-cell infiltration and activation (11). Thus, the limited efficacy of immunotherapy in osteosarcoma is unlikely to be explained solely by insufficient use of immune checkpoint inhibitors, but rather reflects the complex and highly suppressive tumour microenvironment. In this context, TAM-targeted therapy may offer a distinct advantage, not because it bypasses these barriers entirely, but because it directly addresses one of the central suppressive compartments of the osteosarcoma microenvironment. By reducing M2-like polarisation, restoring phagocytic activity, alleviating metabolic and cytokine-mediated immune suppression, and potentially improving T-cell responsiveness, TAM modulation may help convert an immune-resistant tumour ecosystem into one that is more permissive to combination immunotherapy. However, this concept still requires rigorous validation in clinically relevant models and biomarker-guided clinical studies.

Another important limitation is the relatively limited integration of clinical trial evidence in the development of TAM-targeted strategies for osteosarcoma. While several therapeutic approaches—such as CSF-1R inhibitors, immune checkpoint inhibitors, and macrophage-modulating agents—have entered clinical evaluation in other solid tumours, their application in osteosarcoma remains sparse and often confined to early-phase or exploratory studies. Moreover, clinical trials in osteosarcoma frequently involve small patient cohorts, heterogeneous treatment histories, and limited biomarker stratification, making it difficult to draw definitive conclusions regarding efficacy.

In addition, most existing clinical studies have not been specifically designed to assess macrophage-targeted endpoints, and therefore provide limited insight into how TAM modulation contributes to therapeutic outcomes. This gap highlights the need for more rationally designed, biomarker-driven clinical trials that incorporate macrophage-related readouts, such as changes in TAM phenotype, spatial distribution, and interaction with other immune cell populations.

An additional and often underappreciated challenge lies in the biological differences between preclinical model systems and human macrophage biology. Most current evidence for TAM-targeted strategies is derived from murine models or *in vitro* experiments, which do not fully recapitulate the complexity of human immune responses. For example, significant interspecies differences exist in macrophage ontogeny, receptor expression profiles, cytokine responsiveness, and metabolic regulation. In addition, widely used murine osteosarcoma models, particularly subcutaneous xenografts, fail to reproduce the anatomical architecture, immune composition, and evolutionary dynamics of human tumours.

In clinical settings, macrophage populations are considerably more heterogeneous and are influenced by patient-specific factors, including prior chemotherapy, systemic immune status, age, and comorbidities. Moreover, the expression and functional relevance of therapeutic targets—such as CSF-1R, PD-L1, and metabolic regulators—may differ substantially between mice and humans, potentially leading to discrepancies in both efficacy and toxicity profiles. These differences may partly explain why strategies that demonstrate robust activity in preclinical models often show limited or inconsistent benefit in clinical trials.

Therefore, improving the translational relevance of TAM-targeted therapies will require increased reliance on human-based systems, including patient-derived samples, organoid models, and advanced single-cell and spatial profiling approaches, to more accurately define macrophage states and therapeutic responses in osteosarcoma.

A third and equally important challenge concerns the practical translation of advanced therapeutic products. Strategies involving gene-modified cell therapies, mRNA-based delivery systems, biomimetic nanocarriers or multifunctional nanoparticles are scientifically innovative, but their path to clinical implementation is substantially more complex than that of conventional small molecules or repurposed drugs. These products may require stringent control of raw materials, reproducible large-scale manufacturing, sterility assurance, batch consistency, storage stability and release specifications. In addition, the route of administration, dosing schedule, biodistribution, organ retention and immune-related toxicities must all be carefully characterised before clinical use. Advanced therapeutic systems cannot be assumed to be clinically translatable based solely on antitumour efficacy demonstrated in mouse models. From a regulatory perspective, highly engineered products often face additional uncertainty regarding classification, quality control standards and long-term safety monitoring. Therefore, future development of TAM-targeted strategies should not focus exclusively on mechanistic innovation, but also on manufacturability, regulatory feasibility and integration into realistic clinical workflows.

Taken together, the future clinical value of TAM-targeted therapy in osteosarcoma will depend not only on whether macrophage reprogramming can inhibit tumour progression biologically, but also on whether such interventions can be biomarker-guided, mechanistically justified in the context of past immunotherapy limitations, and practically deliverable under real-world manufacturing and regulatory constraints. These considerations are essential for bridging the gap between experimental promise and clinically meaningful application.

## 6 Conclusion and perspective

Osteosarcoma is a highly invasive and easily metastatic malignant bone tumour that has long posed significant treatment challenges, particularly with regard to metastasis and recurrence. Although traditional strategies combining surgery and chemotherapy have significantly improved survival rates for patients with early-stage disease, they are less effective for those with advanced or metastatic disease. This has prompted a shift in research towards the critical area of the tumour microenvironment. In recent years, TAM has become a key breakthrough in understanding the progression mechanisms of osteosarcoma and in developing novel therapies, as it is one of the most important immune infiltrating cells. Here, we systematically elucidate the role of macrophage polarisation in the development and progression of osteosarcoma. Overall, TAMs convert into the pro-tumour M2 phenotype under the stimulation of specific microenvironments (such as IL-4 and IL-13). This subsequently drives the malignant progression of osteosarcoma through a network mechanism that promotes growth and proliferation, immune evasion, angiogenesis and distant metastasis. In terms of growth and reproduction, TAM of the M2 type can stimulate the proliferation of osteosarcoma and resist apoptotic signals by directly secreting growth factors, such as EGF and FGF, or by delivering exosomes that activate the AKT signalling pathway. In the context of immune evasion, M2-like TAMs directly inhibit the function of T and NK cells by expressing high levels of ligands such as CD47 and PD-L1, and by secreting immunosuppressive factors such as TGF-β and IL-10. They also recruit Tregs, collectively constructing an immunosuppressive microenvironment that enables tumour cells to evade immune surveillance. Furthermore, TAM of the M2 type can secrete IL-18, IL-22 and MMPs, which effectively induce EMT and degrade the extracellular matrix. Simultaneously, they strongly promote tumour angiogenesis by releasing VEGF, PDGF-B and specific exosomes, which provide nutritional support for the rapid growth of primary and metastatic lesions.

In light of the above mechanisms, this review categorises intervention strategies innovatively into three main areas: 1) repolarising M2-like TAMs to M1-like, 2) inducing naïve/unpolarised TAMs to polarise towards M1-like, and 3) inhibiting the conversion of M1-like to M2-like. Specific therapeutic strategies include novel cytokine inhibitors such as CSF-1R inhibitors (e.g. PLX3397), combined applications of immune checkpoint inhibitors (e.g. anti-PD-1) with chemotherapeutic agents, natural products (e.g. β-caryophyllene), emerging nanomaterials (e.g. manganese-containing nanoparticles) and sonodynamic therapy. These strategies have demonstrated significant potential for synergistic enhancement in preclinical studies, signalling a shift in osteosarcoma treatment from the traditional ‘cytotoxic’ model to a more precise approach involving ‘microenvironment remodelling’ and ‘immune reprogramming’.

Despite progress in osteosarcoma treatment targeting macrophage polarisation, many issues remain to be addressed. Firstly, the regulatory network of macrophage polarisation has not yet been fully elucidated. The mechanisms by which cytokines, signalling pathways (such as NF-κB and JAK-STAT) and metabolic reprogramming interact still require in-depth analysis. A crucial direction for future research is the application of single-cell sequencing and spatial transcriptomics to dissect the functional heterogeneity of TAM subtypes and their interactions with other immune and stromal cells in osteosarcoma biopsies, both pre- and post-therapy. This will provide critical insights into how TAMs are dynamically modulated by the TME and how these changes correlate with treatment outcomes.

Secondly, most existing intervention strategies are still in the preclinical research stage. The efficiency of targeted drug delivery, *in vivo* stability and long-term safety still need to be validated. In this regard, developing ‘smart’ drug delivery systems that can respond to specific TME cues, such as matrix metalloproteinase (MMP) activity or low pH, to achieve spatially and temporally controlled macrophage reprogramming is a promising area of research. These systems could ensure that therapeutic interventions are directed precisely to the tumour microenvironment, thereby maximising efficacy while minimising systemic side effects.

A third direction for future translational research is the design of humanized mouse models incorporating patient-derived xenografts (PDX) and immune systems to better test combination immunotherapies. These models would more accurately mimic the human osteosarcoma TME, allowing for better prediction of clinical outcomes. Furthermore, building a more comprehensive prognosis prediction model based on TAM phenotypes and their interactions with other TME components will enable more precise, individualized treatment plans.

Lastly, interdisciplinary collaboration and the development of clinical-grade, manufacturable solutions are essential to translating these findings into real-world therapies. The substantial practical and regulatory challenges of manufacturing and administering advanced therapeutic products (ATMPs), such as gene-modified cell therapies or complex nanoparticles, cannot be overlooked. Future research must also focus on overcoming the barriers to large-scale production, stability, regulatory approval, and delivery to ensure these therapies can transition from the lab to the clinic.

In summary, while the therapeutic targeting of TAMs in osteosarcoma is a promising strategy, future research must focus on elucidating the complex regulatory networks of macrophage polarisation, developing clinically applicable biomarker systems, overcoming technical and regulatory challenges in drug delivery, and designing more relevant preclinical models. Addressing these critical issues will pave the way for more effective and personalized treatments for osteosarcoma, ultimately improving patient outcomes.
